# Progressive Gait Deficits in Parkinson’s Disease: A Wearable-Based Biannual 5-Year Prospective Study

**DOI:** 10.3389/fnagi.2019.00022

**Published:** 2019-02-13

**Authors:** Markus A. Hobert, Susanne Nussbaum, Tanja Heger, Daniela Berg, Walter Maetzler, Sebastian Heinzel

**Affiliations:** ^1^Department of Neurology, Christian-Albrechts-University zu Kiel, Kiel, Germany; ^2^Department of Neurodegenerative, Hertie Institute for Clinical Brain Research, University of Tübingen, Tübingen, Germany; ^3^German Center for Neurodegenerative Diseases (DZNE), Tübingen, Germany

**Keywords:** Parkinson’s disease, progression marker, gait, wearable sensor, prospective study

## Abstract

**Background:** Gait changes occur during all Parkinson’s disease (PD) stages and wearable sensor-derived gait parameters may quantify PD progression. However, key aspects that may qualify quantitative gait parameters as progression markers in PD remain elusive.

**Objectives:** Longitudinal changes in gait parameters from a lower-back sensor under convenient and challenging walking conditions in early- and mid-stage PD patients (E-PD, M-PD) compared to controls were investigated.

**Methods:** Normal- and fast-pace parameters (step: number, time, velocity, variability) were assessed every 6 months for up to 5 years in 22 E-PD (<4 years baseline disease duration), 18 M-PD (>5 years) and 24 controls. Parameter trajectories and associations with MDS-UPDRS-III were tested using generalized estimating equations.

**Results:** Normal-pace step number (annual change in E-PD: 2.1%, Time^∗^Group: *p* = 0.001) and step time variability (8.5%, *p* < 0.05) longitudinally increased in E-PD compared to controls (0.7%, -12%). For fast pace, no significant progression differences between groups were observed. Longitudinal changes in M-PD did not differ significantly from controls. MDS-UPDRS-III was largely associated with normal-pace parameters in M-PD.

**Conclusion:** Wearables can quantify progressive gait deficits indicated by increasing step number and step time variability in E-PD. In M-PD, and for fast-pace, gait parameters possess limited potential as PD progression markers.

## Introduction

Progression markers in Parkinson’s disease (PD) are key to advances in PD prognosis and novel treatment efficacy measures. Yet, objective, reliable and quantitative markers of progressive motor deficits are still largely missing. Commonly, semiquantitative rating scales such as the MDS-UPDRS (Goetz et al., 2007) are used to assess motor symptoms and effects of disease modifyers. However, such clinical ratings are to some extent subjective, substantially placebo-responsive (Shin et al., 2016), partly rater-dependent (Post et al., 2005) and therefore prone to bias. Previously, stopwatch-based motor performance measures have been suggested as progression markers, specifically “turning pegs” and “inserting pegs” in functional dexterity/pegboard tests as measures of upper extremity brady- and hypokinesia (Haaxma et al., 2010). These timed measures have been shown to worsen significantly in early-stage (E-PD) but not mid-stage PD (M-PD) patients over 4 years compared to controls (Heinzel et al., 2017). For timed axial measures, including gait speed and timed-up-and-go-test, progression differences were not significant. However, using wearable sensors (so-called “wearables”) gait can be quantified more specifically and more precisely suggesting promising potential of wearables-based progression markers. Previously, quantitative gait parameters have been prospectively assessed in de-novo PD patients (Galna et al., 2015). While not compared to healthy controls (HC), step length and swing time during convenient gait significantly decreased in PD from baseline to month 18. A subsequent 36-month analysis also including HC showed significant group differences between time points regarding step time, length and width variability (Rochester et al., 2017). One other study with unstandardized follow-up intervals compared gait parameters with the change of the item “gait” of the MDS-UPDRS-III and found an association between the worsening in the item “gait” and a decrease of stride length (Schlachetzki et al., 2017). However, key aspects that may qualify these quantitative gait parameters as progression markers in PD remain elusive. In particular, short interval progression characteristics over longer periods, progression in M-PD, and unspecific longitudinal changes in HC need further investigation. Moreover, whether the assessment of gait under convenient or challenging conditions best reveals progressive gait deficits in PD is still unknown.

The present prospective longitudinal study therefore investigated normal- and fast-pace gait as assessed with a lower-back wearable and a validated algorithm deriving gait parameters in E-PD, M-PD, and HC. Assessments were performed 6-monthly for up to 5 years. Differences in longitudinal changes of gait parameters in the PD groups relative to HC were analyzed.

## Materials and Methods

### Prospective Study Design and Participants

Prospective data of the MODEP study (MODeling Epidemiological data to study Parkinson’s disease progression) (Heinzel et al., 2016, 2017) with standardized biannual clinical and gait assessments over up to 5 years (10 visits) were analyzed. Forty patients with PD according United Kingdom Brain Bank criteria (Hughes et al., 1993), and 24 age- and sex-matched HC were included. Since symptom progression can depend on PD duration (Haaxma et al., 2010), patients were recruited as and *a priori* stratified into E-PD (<4 years baseline disease duration, *n* = 22) and M-PD (>5 years, *n* = 18) as suggested by neuropathological findings (Kordower et al., 2013). The study was approved by the local ethical committee (University of Tübingen; No 46/2010). All participants gave written informed consent.

### Clinical Assessment

Clinical assessments comprised current medication, height, weight, clinical ratings of PD motor symptoms (MDS-UPDRS-III) (Goetz et al., 2007), and Hoehn and Yahr stage. For axial scores, MDS-UPDRS-III axial items (3.9/3.10/3.12/3.13/3.14) were summed (Levy et al., 2000). Levodopa equivalent daily dose [LEDD; mg/day] was calculated (Tomlinson et al., 2010). Visits differed in ON/OFF medication state (E-PD: 18%; M-PD: 25% of visits in ON state) which was accounted for in statistical analyses. Moreover, the freezing of gait (FOG) questionnaire (Giladi et al., 2009) and the Montreal Cognitive Assessment (MoCA) (Nasreddine et al., 2005) were assessed. For FOG a score of 3 or higher in item #3, and for mild cognitive impairment a MoCA score of 22 or lower scores were considered (Carson et al., 2018).

### Gait Assessment

Participants were instructed to walk 20 m, first with normal (convenient) pace and then with fast pace (individual maximum), along a 2 m-wide straight corridor. Both conditions were performed twice, and gait parameters were averaged for the two trials. The wearable (Dynaport Hybrid, McRoberts B.V., The Hague, Netherlands) was fixed with a belt to the participants’ lower back. The Dynaport Hybrid is an inertial measurement unit containing a 3D-accelerometer and a 3D-gyroscope with 100 Hz sampling frequency. Reliability of the sensor system and derived movement parameters has been shown previously using a instrumented Timed Up and Go (TUG) tests (van Lummel et al., 2016). After discarding acceleration and deceleration periods of walks (first and last 15% of the data; about 3 m each) (Lindemann et al., 2008), the company-provided validated gait analysis algorithm (Zijlstra and Hof, 2003; Brandes et al., 2006; Dijkstra et al., 2008; Houdijk et al., 2008; Hobert et al., 2017) was applied to extract the following gait parameters: step number, step time, step velocity, and measures of gait variability, i.e., step time variability (calculated as coefficient of variation), gait asymmetry (Yogev et al., 2007; Plotnik et al., 2009) and phase coordination index (PCI) (Plotnik et al., 2007).

### Statistical Approach

Longitudinal data of gait parameters were analyzed using generalized estimating equations (GEE) with identity-link functions with normal distributions and exchangeable working correlation structure (Zeger et al., 1988; Hardin and Hilbe, 2003). GEE models comprised the subject ID, the within-subject variable Time (visit 1 to 10; centered), the factor Group (E-PD vs. HC; M-PD vs. HC), the interaction term Time^∗^Group (i.e., group difference in progression) and the covariates age (at baseline), ON/OFF medication state, weight, height, body-mass-index were considered. Parameters for normal- and fast-pace gait were selected as dependent variables. Group effects are related to the median of the observational period. The significance level was α = 5% (two-sided). Bonferroni-corrections for multiple testing were applied considering two group comparisons and two gait conditions (*p* < 0.0125, significance threshold; 0.0125 < *p* < 0.05, statistical trend). Moreover, gait parameters were tested for associations with clinical PD parameters (MDS-UPDRS-III total score, axial score, Hoehn and Yahr stage, LEDD). Here, GEE models were calculated for the overall PD sample, and separately for E-PD and M-PD, comprising the respective clinical parameter and aforementioned covariates (except Group). For these exploratory analyses no correction for multiple testing was considered (*p* < 0.05). We used IBM SPSS Statistics, V22.0 (Armonk, NY, IBM Corp.) for statistical analyses.

## Results

E-PD and M-PD did not differ significantly in age and sex-ratio compared to HC. M-PD differed significantly in disease duration, but also in LEDD, MDS-UPDRS-III scores and Hoehn and Yahr stage (Supplementary Table S1).

PD groups showed a significantly larger step number and lower velocity compared to HC for normal-pace (E-PD: *p* = 0.001; M-PD: *p* < 0.001) and for fast-pace gait (*p* < 0.001). For GEE analyses, see Supplementary Tables S2, S3. Moreover, normal-pace step time CoV was significantly higher in M-PD compared to HC (*p* = 0.002). None of the other gait parameters showed significant differences between the PD groups and HC (*p* > 0.05), neither for normal- nor for fast-pace gait.

**FIGURE 1:**
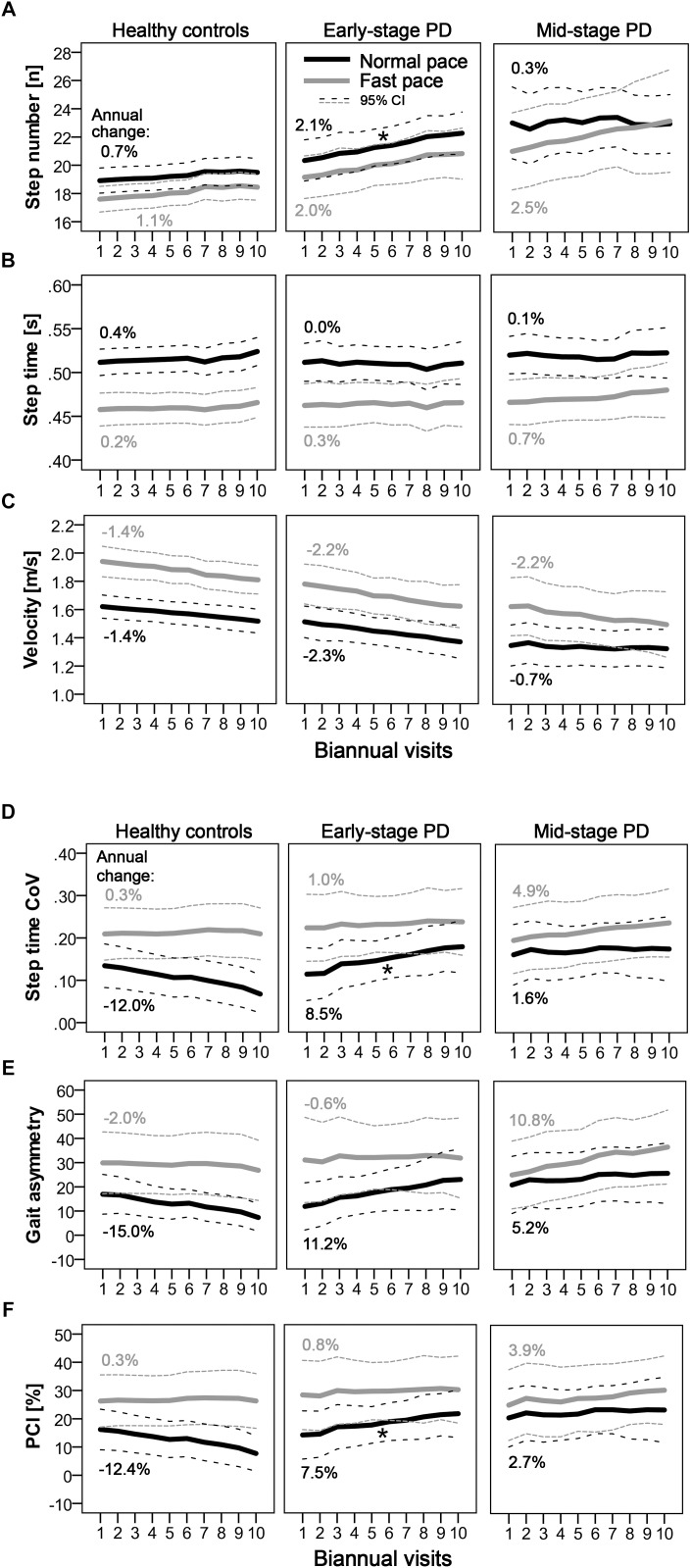
Longitudinal progression of quantitative gait characteristics as indicated by step number **(A)** step time **(B)** velocity **(C)** and parameters of gait variability. **(D–F)** Mean values per visit and 95% confidence intervals are shown for normal pace (black) and fast pace (gray) conditions for PD groups (early-stage and mid-stage) and controls. Annual changes [%] of gait parameters are indicated. Asterisks denote significant (Time^∗^Group: *p* < 0.0125; Bonferroni-corrected) differences in longitudinal changes compared to controls.

Longitudinal changes of the quantitative gait parameters are shown in Figure 1. Significant differences in longitudinal changes (*p* < 0.0125; Bonferroni-corrected) were only observed between HC and E-PD, and only for normal pace. Specifically, the progression in normal-pace step number differed significantly between E-PD and HC (Time^∗^Group, *p* = 0.001), with a significant increase over time only in E-PD (Time: *p* < 0.001), but not HC (Time: *p* = 0.070). Moreover, E-PD and HC differed regarding the longitudinal changes in normal-pace step time CoV (Time^∗^Group, *p* = 0.002), gait asymmetry (*p* = 0.009), and for trend, PCI (*p* = 0.028). However, while HC showed a decrease over time in gait variability parameters (step time CoV, *p* < 0.001; gait asymmetry, *p* = 0.003; PCI, *p* = 0.015), no significant change over time was observed in E-PD (*p* > 0.05). For fast-pace, none of the longitudinal changes of gait parameters differed significantly between groups.

Excluding individuals who at least once fulfilled the criteria for mild cognitive impairment (3 HC, 5 E-PD, 2 M-PD) or entering MoCA scores as additional factor into GEE analyses largely did not change the main results. Similarly, excluding individuals who reported freezing of gait (2 E-PD, 4 M-PD) or excluding visits in ON medication state resulted in the main findings of progression group differences in normal-pace step number and step time CoV, which remained significant and showed similar effect sizes. To further test the reliability of the results, we analyzed the performances in single gait trials instead of the average of the two trials. For each of the two single trials the interaction effect (Time^∗^Group) was observed (*p* < 0.05) for normal-pace step number and step time CoV in E-PD versus HC comparisons. Analyses of the ratio of normal/fast-pace gait parameters showed the longitudinal changes of ratio values to be less pronounced and with larger variability between visits and individuals compared to normal-pace parameters alone.

Associations between clinical parameters and wearables-based gait parameters partly differed between gait conditions (normal/fast-pace) and PD groups (Table 1). For normal pace, gait variability in E-PD, and in M-PD additionally step number and velocity showed significant associations with clinical parameters. For fast pace, E-PD but not M-PD showed associations of gait variability with clinical parameters. In M-PD, axial scores showed larger associations with fast-pace compared to normal-pace step number and velocity.

**Table 1:** Associations of wearables-based and clinical parameters of gait deficits.

Gait condition	Clinical parameter (defined unit)	All PD patients	Relative and absolute change per 1-unit change of clinical parameter (mean ± S.E.)	E-PD	Relative and absolute change per 1-unit change of clinical parameter (mean ± S.E.)	M-PD	Relative and absolute change per 1-unit change of clinical parameter (mean ± S.E.)
Normal pace	**MDS-UPDRS-III**	Step number	3%, 0.67 ± 0.19			Step number	4%, 0.80 ± 0.24
	(Unit: 10 scores of	Velocity	-3%, -0.04 ± 0.01			Velocity	-3%, -0.04 ± 0.01
	max. 120)	Step time CoV	14%, 0.02 ± 0.01			Step time CoV	13%, 0.02 ± 0.01
		Gait asym.	18%, 3.71 ± 1.36			Gait asym.	18%, 4.34 ± 1.95
		PCI	17%, 3.38 ± 1.16			PCI	15%, 3.40 ± 1.19
	**AXIAL**	Step number	8%, 1.84 ± 0.43	Step time CoV	47%, 0.07 ± 0.03	Step number	10%, 2.21 ± 0.51
	(Unit: 5 scores of	Velocity	-7%, -0.09 ± 0.02	PCI	53%, 9.19 ± 3.93	Velocity	-8%, -0.11 ± 0.02
	max. 20)	Step time CoV	33%, 0.05 ± 0.01			Step time CoV	29%, 0.05 ± 0.01
		Gait asym.	44%, 8.87 ± 2.27			Gait asym.	46%, 10.92 ± 4.18
		PCI	36%, 7.14 ± 2.27			PCI	32%, 7.13 ± 2.63
	**H and Y**	Step time CoV	26%, 0.04 ± 0.01	Gait asym.	27%, 4.59 ± 1.93	Step time CoV	25%, 0.04 ± 0.01
	(Unit: 1 stage of	Gait asym.	34%, 6.91 ± 1.56			Gait asym.	34%, 8.06 ± 2.66
	max. 5)	PCI	29%, 5.79 ± 1.60			PCI	28%, 6.27 ± 1.27
	**LEDD**			Step time CoV	8%, 0.01 ± 0.00		
	(Unit: 100 mg/day)			PCI	7%, 1.25 ± 0.60		

Fast pace	**MDS-UPDRS-III**	Step number	3%, 0.66 ± 0.19	CoV	-7%, -0.02 ± 0.01	Step number	5%, 1.04 ± 0.31
	(Unit: 10 scores of	Velocity	-3%, -0.04 ± 0.01	Gait asym.	-10%,	Velocity	-4%, -0.06 ± 0.02
	max. 120)				-3.05 ± 1.44		
	**AXIAL**	Step number	14%, 2.89 ± 0.75			Step number	17%, 3.64 ± 0.95
	(Unit: 5 score of max. 20)	Velocity	-9%, 0.16 ± 0.03			Velocity	-13%, -0.20 ± 0.04
	**H and Y**	Step number	5%, 1.07 ± 0.33			Step number	8%, 1.74 ± 0.55
	(Unit: 1 stage of max. 5)					Velocity	-6%, -0.10 ± 0.05
	**LEDD**	Step number	1%, 0.21 ± 0.10	Step time CoV	9%, 0.02 ± 0.01		
	(Unit: 100 mg/day)			PCI	9%, 2.74 ± 1.04		


*Significant associations (p < 0.05) between clinical measures of Parkinson’s disease and wearables-based gait parameters are shown. Upper part: normal-pace parameters; lower part: fast-pace parameters. The parameter name and the relative (in %) and the absolute change of the parameter that occurred with one unit change of the respective clinical measure (shown on the left) are indicated. In columns, analysis results are shown for the entire PD group (left), and when only considering E-PD or M-PD groups. H and Y, Hoehn and Yahr stage; CoV, coefficient of variance; E-PD, early-stage Parkinson’s disease; gait asym., gait asymmetry; LEDD, levodopa equivalent daily dose; MDS-UPDRS-III, Movement Disorder Society sponsored Unified Parkinson’s Disease Rating Scale, part 3, motor examination; M-PD, mid-stage Parkinson’s disease; PCI, phase coordination index; S.E., standard error.*

## Discussion

The present prospective observational study with 6-month intervals over a period of up to 5 years investigated the potential of quantitative gait parameters for the assessment of changes in normal- and fast-pace gait, respectively, in early- and mid-stage PD patients.

This study supports and extends previous findings suggesting longitudinal changes of normal-pace step number as a potential progression marker in E-PD (Galna et al., 2015; Schlachetzki et al., 2017). Changes were significantly larger in E-PD (2.1%/year) than in HC (0.7%/year), and, importantly, showed linear progression over the 5-year observation period. These findings remained robust when also accounting for further potential confounders and when excluding individuals with mild cognitive impairment and freezing of gait. This makes step number during normal pace a very promising and robust progression parameter for routine diagnostics and clinical trials.

Gait variability parameters may also possess potential as a progression marker in early phases of clinical PD. Significant differences in longitudinal changes were observed between E-PD and HC, however, this finding was not (only) due to a (non-significant) increase in PD (8.5%/year) but partly driven by HC decreasing in gait variability over time. Our finding supports our clinical impression that variability of gait shows relevant changes in short time periods in many PD patients, and may be one of the best predictors of disease milestones like falls (Hausdorff et al., 2003; Callisaya et al., 2011; Henderson et al., 2016). Indeed, previous cross-sectional analyses showed increased gait variability to be associated with PD duration (Hausdorff et al., 2003). Thus, the potential as PD progression marker should be further evaluated in future studies.

M-PD did not show any significant gait changes compared to HC during the relatively long observation period. With increasing PD duration between-patient differences in PD severity and phenotypes might become more apparent, thus single PD progression markers might not be valid for all PD patients (Fereshtehnejad et al., 2015). Overall, our results argue against a relevant potential of quantitative gait analysis for the evaluation of disease progression in mid-stage PD.

Interestingly, normal-pace walking was more sensitive to gait-related changes than fast-pace walking. Compared to normal-pace, fast-pace gait might be more challenging and stressful, which may increase (behavioral) variability and confounding with unspecific age-related factors. Therefore, we recommend normal-pace walking to be included in, e.g., clinical trials to assess PD progression, and to omit challenging walking conditions and normal/fast-pace comparisons.

Established clinical PD parameters showed associations with the quantitative wearables-based gait parameters thereby supporting their value and clinical relevance. Associations were largely observed in M-PD, and for normal-pace conditions. Differences in associations might be due to lower variance of the clinical data in E-PD than in M-PD. Moreover, MDS-UPDRS-III total and axial sum scores may less specifically indicate motor symptoms compared to quantitative gait parameters, and in E-PD more specific quantification of motor symptoms may be required. Fast-pace conditions might introduce unspecific (error) variance into gait variability parameter data, thus compared to normal-pace their associations with clinical measures might be weaker.

These present results might also provide future clinical and scientific perspectives. Parameters that allow to quantify disease progression, such as kinetic gait parameters derived from wearable devices, could serve as objective outcome markers of pharmacological treatment and other interventions. In this regard reliable quantitative markers of PD motor symptoms and their progression are highly promising as (at least secondary) outcomes in the clinical routine as well as in clinical intervention trials (Moreau et al., 2018). These measures have the potential of increasing sensitivity and objectivity while reducing costs of these clinical trials (Merchant et al., 2018). Importantly, wearable-based motor measures possess a high relevance for daily living and quality of life (Van Uem et al., 2016), and studies based on such measures may therefore yield results that benefit PD patients.

This study faces some limitations. First, the sample was relatively small. However, the thorough and high-frequency evaluation of participants may partially compensate for this limitation, especially as parameters are needed that are robust even in smaller samples. Second, PD is a heterogeneous disease and further subgroups and non-motor symptoms potentially influencing gait, e.g., depression, were not accounted for. Also, multi-facetted interventions were not controlled for in this observational study. Longitudinal differences in pharmacological and non-pharmacological treatments and their efficacy over time may additionally contribute to the heterogeneity in PD, particularly in advanced PD. While accounting for ON/OFF medication state in statistical analyses modeling of individual treatments is complex. Thus, subtle gait changes and factors contributing to progression differences between PD patients might have remained undetected.

To conclude, number of steps and possibly also gait variability measures as assessed during normal-pace walking with a lower-back wearable are robust and promising progression markers in the early phase of PD.

## Author Contributions

DB and WM conceived the study. DB, WM, TH, and SN organized the study. WM, TH, SN, and MH executed the study. SH and MH designed the statistical analysis. SH executed the statistical analysis. DB, WM, and MH critically reviewed the results. MH and SH wrote the first draft of the manuscript. DB, WM, and MH critically reviewed the manuscript.

## Conflict of Interest Statement

The authors declare that the research was conducted in the absence of any commercial or financial relationships that could be construed as a potential conflict of interest.
