# Supplementary material for: Progressive Gait Deficits in Parkinson’s Disease: A Wearable-Based Biannual 5-Year Prospective Study
Source: Front Aging Neurosci. 2019 Feb 13;11:22. doi: 10.3389/fnagi.2019.00022 (PMC6381067; doi:10.3389/fnagi.2019.00022)
Supplement: Supplementary file 1 [file Data_Sheet_1.PDF]

***Supporting Material:*** *Progressive gait deficits in Parkinson's disease: A wearable-based biannual 5-year prospective study*

## Supporting Table 1: Clinical and demographic variables

| | **Healthy controls** | | **Early-stage PD** | | **Mid-stage PD** | | **p-values** |
|---|---|---|---|---|---|---|---|
| | Mean ± S.D. | Range | Mean ± S.D. | Range | Mean ± S.D. | Range | E-PD/HC<br>M-PD/HC<br>E-PD/M-PD |
| Number of subjects | 24 | | 22 | | 18 | | |
| Age at visit 1 [years] | 63 ± 7 | 51 - 76 | 62 ± 8 | 41 - 73 | 67 ± 5 | 57 - 77 | .45<br>.08<br>**.02** |
| Sex [females/males] | 10 / 14 | | 9 / 13 | | 7 / 11 | | .60<br>.55<br>.58 |
| Disease duration at visit 1 [years] | | | 1.5 ± 1.0 | 0.1 – 3.4 | 6.6 ± 1.1 | 4.3 - 8.1 | n.a.<br>n.a.<br>**<.01** |
| Age at diagnosis [years] | | | 60 ± 8 | 40 - 72 | 60 ± 5 | 51 - 71 | n.a.<br>n.a.<br>.94 |
| LEDD at visit 1 [mg/d] | | | 236 ± 163 | 52 - 607 | 695 ± 343 | 80 - 1378 | n.a.<br>n.a.<br>**<.01** |
| MDS-UPDRS-III at visit 1 | 1.6 ± 1.7 | 0 - 6 | 21 ± 8 | 5 - 32 | 35 ± 14 | 8 - 68 | **<.01**<br>**<.01**<br>**<.01** |
| Hoehn & Yahr stage at visit 1 | | | 1.7 ± 0.6 | 1 - 3 | 2.4 ± 0.7 | 1 - 4 | n.a.<br>n.a.<br>**<.01** |
| MoCA at visit 1 | 27.0 ± 1.9 | 23 - 30 | 26.0 ± 2.8 | 19 - 29 | 26.5 ± 1.9 | 23 - 30 | .30<br>.33<br>.95 |
| FOG [number of freezers, any visit] | | | 2 / 22 | | 4 / 18 | | n.a.<br>n.a.<br>.52 |

FOG, Freezing of Gait; LEDD, levodopa equivalent daily dose; MDS-UPDRS-III, Movement Disorder Society sponsored Unified Parkinson's Disease Rating Scale, part 3, motor examination; MoCA, Montreal Cognitive Assessment; n.a., not applicable; PD, Parkinson's disease; S.D., standard deviation. Fisher exact tests for differences in sex ratios and FOG, t-tests of independent groups and, for differences in Hoehn & Yahr stages, Mann-Whitney U-tests were calculated. Significant p-values (p<.05) are bold.

## Supporting Table 2: Longitudinal analyses of normal pace wearables gait parameters

| | | E-PD vs. HC | | | M-PD vs. HC | | |
|---|---|---|---|---|---|---|---|
| | GEE parameter | B | S.E. | *P*-value | B | S.E. | *P*-value |
| Step number [n] | Constant | 66.280 | 36.9012 | .072 | 4.317 | 39.0746 | .912 |
| | Group [-0.5, 0.5] | 2.110 | 0.4920 | **<.001** | 4.323 | 0.9441 | **<.001** |
| | Time$^x$ (6-month change in PD) | 0.218 | 0.0291 | **<.001** | 0.036 | 0.0785 | .651 |
| | Time$^y$ (6-month change in HC) | 0.067 | 0.0369 | .070 | 0.064 | 0.0361 | .078 |
| | Time*Group (Progression difference) | -0.151 | 0.0471 | **.001** | 0.028 | 0.0860 | .743 |
| | Age [years] | -0.001 | 0.0378 | .978 | 0.057 | 0.0598 | .342 |
| | ON/OFF medication [-0.5, 0.5] | 0.028 | 0.2149 | .897 | -1.728 | 0.7059 | .014 |
| | Weight [kg] | 0.226 | 0.2389 | .344 | -0.173 | 0.2415 | .474 |
| | Height [m] | -27.575 | 21.1779 | .193 | 8.979 | 22.0394 | .684 |
| | BMI | -0.574 | 0.6859 | .403 | 0.516 | 0.7503 | .492 |
| Step time [s] | Constant | -0.780 | 0.5091 | .125 | -0.616 | 0.4614 | .182 |
| | Group [-0.5, 0.5] | -0.006 | 0.0082 | .456 | -0.005 | 0.0030 | .562 |
| | Time$^x$ (6-month change in PD) | 0.000 | 0.0009 | .914 | 0.000 | 0.0012 | .893 |
| | Time$^y$ (6-month change in HC) | 0.001 | 0.0006 | .071 | 0.001 | 0.0006 | .078 |
| | Time*Group (Progression difference) | 0.001 | 0.0011 | .353 | 0.001 | 0.0014 | .519 |
| | Age [years] | 0.001 | 0.0006 | .063 | 0.002 | 0.0006 | **.006** |
| | ON/OFF medication [-0.5, 0.5] | 0.003 | 0.0047 | .549 | 0.007 | 0.0086 | .393 |
| | Weight [kg] | -0.006 | 0.0032 | .086 | -0.005 | 0.0030 | .099 |
| | Height [m] | 0.733 | 0.2928 | **.012** | 0.604 | 0.2789 | .030 |
| | BMI | 0.015 | 0.0098 | .123 | 0.014 | 0.0088 | .111 |
| Velocity [m/s] | Constant | 2.013 | 2.5777 | .435 | 4.272 | 2.6794 | .111 |
| | Group [-0.5, 0.5] | -0.135 | 0.0405 | **.001** | -0.239 | 0.0591 | **<.001** |
| | Time$^x$ (6-month change in PD) | -0.017 | 0.0037 | **<.001** | -0.004 | 0.0052 | .388 |
| | Time$^y$ (6-month change in HC) | -0.011 | 0.0034 | **.002** | -0.011 | 0.0034 | **.002** |
| | Time*Group (Progression difference) | 0.006 | 0.0051 | .252 | -0.006 | 0.0062 | .328 |
| | Age [years] | -0.003 | 0.0029 | .252 | -0.007 | 0.0038 | .056 |
| | ON/OFF medication [-0.5, 0.5] | -0.009 | 0.0238 | .714 | 0.082 | 0.0305 | **.008** |
| | Weight [kg] | 0.001 | 0.0161 | .940 | 0.016 | 0.0162 | .313 |
| | Height [m] | -0.218 | 1.4649 | .882 | -1.474 | 1.5426 | .339 |
| | BMI | -0.003 | 0.0494 | .947 | -0.046 | 0.0489 | .346 |
| Step time CoV | Constant | -0.115 | 1.3747 | .933 | 1.928 | 1.5416 | .211 |
| | Group [-0.5, 0.5] | 0.039 | 0.0218 | .071 | 0.078 | 0.0256 | **.002** |
| | Time$^x$ (6-month change in PD) | 0.006 | 0.0038 | .109 | 0.001 | 0.0044 | .760 |
| | Time$^y$ (6-month change in HC) | -0.007 | 0.0019 | **.000** | -0.007 | 0.0020 | **.001** |
| | Time*Group (Progression difference) | -0.013 | 0.0042 | **.002** | -0.008 | 0.0048 | .092 |
| | Age [years] | -0.004 | 0.0016 | **.011** | -0.006 | 0.0021 | **.002** |
| | ON/OFF medication [-0.5, 0.5] | -0.020 | 0.0191 | .286 | 0.030 | 0.0285 | .292 |
| | Weight [kg] | -0.005 | 0.0089 | .559 | 0.008 | 0.0098 | .416 |
| | Height [m] | 0.182 | 0.7950 | .819 | -0.877 | 0.8774 | .318 |
| | BMI | 0.023 | 0.0267 | .381 | -0.017 | 0.0298 | .558 |
| Gait asymmetry | Constant | -432.454 | 297.866 | .146 | 127.698 | 2.988.304 | .669 |
| | Group [-0.5, 0.5] | 5.110 | 35.294 | .148 | 13.631 | 48.971 | **.005** |
| | Time$^x$ (6-month change in PD) | 0.976 | 0.7928 | .218 | 0.584 | 0.5759 | .311 |
| | Time$^y$ (6-month change in HC) | -1.039 | 0.4284 | .015 | -1.067 | 0.4527 | .018 |
| | Time*Group (Progression difference) | -2.015 | 0.9149 | .028 | -1.650 | 0.7315 | .024 |
| | Age [years] | -0.684 | 0.1827 | **<.001** | -0.912 | 0.3392 | **.007** |
| | ON/OFF medication [-0.5, 0.5] | -1.954 | 27.185 | .472 | 4.264 | 39.169 | .276 |
| | Weight [kg] | -3.581 | 19.395 | .065 | -0.005 | 18.024 | .998 |
| | Height [m] | 264.063 | 168.9376 | .118 | -40.126 | 165.8789 | .809 |
| | BMI | 12.102 | 61.176 | .048 | 0.968 | 54.866 | .860 |
| PCI [%] | Constant | -96.998 | 182.0451 | .594 | 149.003 | 223.0506 | .504 |
| | Group [-0.5. 0.5] | 4.951 | 30.246 | .102 | 11.572 | 34.771 | **.001** |
| | Time$^x$ (6-month change in PD) | 0.647 | 0.4958 | .192 | 0.316 | 0.6732 | .639 |
| | Time$^y$ (6-month change in HC) | -0.864 | 0.2930 | **.003** | -0.854 | 0.2967 | **.004** |
| | Time*Group (Progression difference) | -1.511 | 0.5756 | **.009** | -1.170 | 0.7343 | .111 |
| | Age [years] | -0.642 | 0.2445 | **.009** | -0.918 | 0.2972 | **.002** |
| | ON/OFF medication [-0.5, 0.5] | -2.149 | 28.343 | .448 | 3.970 | 45.812 | .386 |
| | Weight [kg] | -1.252 | 11.753 | .287 | 0.373 | 14.432 | .796 |
| | Height [m] | 74.106 | 105.1493 | .481 | -52.822 | 126.6612 | .677 |
| | BMI | 4.761 | 35.483 | .180 | -0.182 | 43.668 | .967 |

**Supporting Table 2: Progression characteristics of wearables-based gait parameters during normal pace in early-stage and mid-stage Parkinson's disease compared to healthy controls over up to 10 biannual visits.** Statistical analyses using Generalized Estimating Equations (GEE) of the MODEP cohort. The coefficient B indicates the group differences (Group) in gait parameters. Visit-wise 6-month changes (Time) and group differences in 6-month changes (Time*Group) were analyzed. Significant effects (*P*<.0125; Bonferroni-corrected) are bold. [x]GEE analyses with HC as reference category. [y]Derived from separate GEE analyses with the respective PD group as reference category. Abbreviations: B, beta regression coefficient; CoV, coefficient of variance; E-PD. early-stage Parkinson's disease; HC, healthy controls; M-PD. mid-stage Parkinson's disease; PCI, phase coordination index; S.E., standard error.

## Supporting Table 3: Longitudinal analyses of fast pace wearables gait parameters

| | GEE parameter | E-PD vs. HC B | S.E. | *P*-value | M-PD vs. HC B | S.E. | *P*-value |
|---|---|---|---|---|---|---|---|
| Step number [n] | Constant | 79.694 | 44.4874 | .073 | 52.761 | 80.7604 | .514 |
| | Group [-0.5. 0.5] | 2.088 | 0.5144 | **<.001** | 4.115 | 0.8387 | **<.001** |
| | Time$^x$ (6-month change in PD) | 0.192 | 0.0469 | **<.001** | 0.273 | 0.1812 | .131 |
| | Time$^y$ (6-month change in HC) | 0.096 | 0.0302 | **.001** | 0.093 | 0.0300 | **.002** |
| | Time*Group (Progression difference) | -0.096 | 0.0555 | .084 | -0.181 | 0.1831 | .324 |
| | Age [years] | 0.011 | 0.0369 | .773 | 0.082 | 0.0465 | .077 |
| | ON/OFF medication [-0.5, 0.5] | -0.036 | 0.2757 | .897 | -0.137 | 1.1887 | .908 |
| | Weight [kg] | 0.292 | 0.2919 | .317 | 0.149 | 0.5516 | .787 |
| | Height [m] | -36.455 | 25.5541 | .154 | -22.413 | 47.8993 | .640 |
| | BMI | -0.775 | 0.8324 | .352 | -0.339 | 1.5598 | .828 |
| Step time [s] | Constant | -0.332 | 0.6570 | .613 | -0.473 | 0.8769 | .589 |
| | Group [-0.5. 0.5] | 0.006 | 0.0093 | .505 | 0.006 | 0.0095 | .520 |
| | Time$^x$ (6-month change in PD) | 0.001 | 0.0008 | .374 | 0.002 | 0.0015 | .252 |
| | Time$^y$ (6-month change in HC) | 0.001 | 0.0005 | .251 | 0.001 | 0.0005 | .261 |
| | Time*Group (Progression difference) | 0.000 | 0.0009 | .890 | -0.001 | 0.0016 | .456 |
| | Age [years] | 0.001 | 0.0006 | **.026** | 0.002 | 0.0006 | **.001** |
| | ON/OFF medication [-0.5, 0.5] | -0.003 | 0.0038 | .373 | -0.004 | 0.0053 | .482 |
| | Weight [kg] | -0.003 | 0.0042 | .492 | -0.004 | 0.0058 | .479 |
| | Height [m] | 0.426 | 0.3793 | .262 | 0.479 | 0.5221 | .358 |
| | BMI | 0.008 | 0.0127 | .546 | 0.012 | 0.0163 | .467 |
| Velocity [m/s] | Constant | -1.569 | 3.9338 | .690 | 3.379 | 6.3621 | .595 |
| | Group [-0.5. 0.5] | -0.200 | 0.0529 | **<.001** | 0.319 | 0.0678 | **<.001** |
| | Time$^x$ (6-month change in PD) | -0.019 | 0.0042 | **<.001** | -0.018 | 0.0108 | .104 |
| | Time$^y$ (6-month change in HC) | -0.013 | 0.0043 | **.002** | -0.013 | 0.0043 | **.002** |
| | Time*Group (Progression difference) | 0.006 | 0.0060 | .322 | 0.004 | 0.0117 | .706 |
| | Age [years] | -0.006 | 0.0036 | .073 | -0.013 | 0.0043 | **.004** |
| | ON/OFF medication [-0.5, 0.5] | 0.009 | 0.0234 | .710 | 0.082 | 0.0630 | .195 |
| | Weight [kg] | -0.020 | 0.0259 | .446 | 0.012 | 0.0422 | .780 |
| | Height [m] | 2.147 | 2.2362 | .337 | -0.583 | 3.7354 | .876 |
| | BMI | 0.057 | 0.0807 | .477 | -0.035 | 0.1203 | .769 |
| Step time CoV | Constant | 1.019 | 1.8753 | .587 | 1.204 | 2.3795 | .613 |
| | Group [-0.5. 0.5] | 0.017 | 0.0292 | .565 | 0.005 | 0.0319 | .865 |
| | Time$^x$ (6-month change in PD) | 0.001 | 0.0033 | .738 | 0.005 | 0.0030 | .076 |
| | Time$^y$ (6-month change in HC) | 0.000 | 0.0025 | .914 | 0.000 | 0.0025 | .914 |
| | Time*Group (Progression difference) | -0.001 | 0.0042 | .840 | -0.005 | 0.0039 | .202 |
| | Age [years] | -0.002 | 0.0023 | .431 | 0.001 | 0.0024 | .651 |
| | ON/OFF medication [-0.5, 0.5] | -0.004 | 0.0209 | .858 | 0.007 | 0.0250 | .781 |
| | Weight [kg] | 0.002 | 0.0126 | .852 | 0.005 | 0.0154 | .755 |
| | Height [m] | -0.498 | 1.0900 | .648 | -0.664 | 1.3577 | .625 |
| | BMI | 0.000 | 0.0365 | .996 | -0.011 | 0.0476 | .818 |
| Gait asymmetry | Constant | 58.891 | 340.1134 | .863 | 147.169 | 446.6789 | .742 |
| | Group [-0.5. 0.5] | 2.692 | 5.9177 | .649 | 3.778 | 6.1664 | .540 |
| | Time$^x$ (6-month change in PD) | -0.082 | 1.0410 | .937 | 1.515 | 0.5737 | **.008** |
| | Time$^y$ (6-month change in HC) | -0.256 | 0.4902 | .602 | -0.303 | 0.4946 | .540 |
| | Time*Group (Progression difference) | -0.173 | 1.1566 | .881 | -1.818 | 0.7666 | .018 |
| | Age [years] | -0.600 | 0.4517 | .184 | 0.177 | 0.4193 | .673 |
| | ON/OFF medication [-0.5, 0.5] | -3.552 | 3.5361 | .315 | -1.487 | 4.3633 | .733 |
| | Weight [kg] | -0.527 | 2.2283 | .813 | 0.159 | 2.8781 | .956 |
| | Height [m] | -10.947 | 193.7224 | .955 | -83.570 | 254.4978 | .743 |
| | BMI | 2.678 | 6.5291 | .682 | 0.194 | 8.8831 | .983 |
| PCI [%] | Constant | 58.268 | 317.0942 | .854 | 149.306 | 438.1206 | .733 |
| | Group [-0.5. 0.5] | 2.607 | 4.4573 | .559 | 0.036 | 4.6533 | .994 |
| | Time$^x$ (6-month change in PD) | 0.122 | 0.4396 | .781 | 0.608 | 0.4432 | .170 |
| | Time$^y$ (6-month change in HC) | 0.039 | 0.3981 | .921 | 0.037 | 0.3998 | .926 |
| | Time*Group (Progression difference) | -0.083 | 0.5912 | .888 | -0.571 | 0.5984 | .340 |
| | Age [years] | -0.323 | 0.3336 | .334 | 0.093 | 0.3454 | .788 |
| | ON/OFF medication [-0.5, 0.5] | -2.276 | 3.2700 | .486 | 5.812 | 3.6733 | .114 |
| | Weight [kg] | -0.136 | 2.1470 | .949 | 0.511 | 2.7814 | .854 |
| | Height [m] | -19.793 | 186.9991 | .916 | -80.014 | 250.1581 | .749 |
| | BMI | 1.372 | 6.1161 | .822 | -1.178 | 8.6316 | .891 |

**Supporting Table 3: Progression characteristics of wearables-based gait parameters during fast pace in early-stage and mid-stage Parkinson's disease compared to healthy controls over up to 10 biannual visits.** Statistical analyses using Generalized Estimating Equations (GEE) of the MODEP cohort. The coefficient B indicates the group differences (Group) in gait parameters. Visit-wise 6-month changes (Time) and group differences in 6-month changes (Time*Group) were analyzed. Significant effects (*P*<.0125; Bonferroni-corrected) are bold. [x]GEE analyses with HC as reference category. [y]Derived from separate GEE analyses with the respective PD group as reference category. B, beta regression coefficient; BMI, body mass index; CoV, coefficient of variance; E-PD, early-stage PD; HC, healthy controls; M-PD, mid-stage PD; PCI, phase coordination index; S.E., standard error.
